# Evaluating Martial Arts Punching Kinematics Using a Vision and Inertial Sensing System

**DOI:** 10.3390/s21061948

**Published:** 2021-03-10

**Authors:** Karlos Ishac, David Eager

**Affiliations:** School of Mechanical and Mechatronic Engineering, University of Technology Sydney, P.O. Box 123, Broadway, NSW 2007, Australia; David.eager@uts.edu.au

**Keywords:** Taekwondo, martial arts, sensors, biomechanics, motion tracking, sports science, human kinematics, dynamic measurement

## Abstract

Martial arts has many benefits not only in self-defence, but also in improving physical fitness and mental well-being. In our research we focused on analyzing the velocity, impulse, momentum and impact force of the Taekwondo sine-wave punch and reverse-step punch. We evaluated these techniques in comparison with the martial arts styles of Hapkido and Shaolin Wushu and investigated the kinematic properties. We developed a sensing system which is composed of an ICSensor Model 3140 accelerometer attached to a punching bag for measuring dynamic acceleration, Kinovea motion analysis software and 2 GoPro Hero 3 cameras, one focused on the practitioner’s motion and the other focused on the punching bag’s motion. Our results verified that the motion vectors associated with a Taekwondo practitioner performing a sine-wave punch, uses a unique gravitational potential energy to optimise the impact force of the punch. We demonstrated that the sine-wave punch on average produced an impact force of 6884 N which was higher than the reverse-step punch that produced an average impact force of 5055 N. Our comparison experiment showed that the Taekwondo sine-wave punch produced the highest impact force compared to a Hapkido right cross punch and a Shaolin Wushu right cross, however the Wushu right cross had the highest force to weight ratio at 82:1. The experiments were conducted with high ranking black belt practitioners in Taekwondo, Hapkido and Shaolin Wushu.

## 1. Introduction

The martial arts have maintained popularity and importance across human history for their physical and mental benefits [1,2]. Amongst martial arts is a style known as Taekwondo, recognized for its emphasis on kicking techniques and bare hand and feet free-style fighting. Taekwondo is a martial art style that originated in Korea over 2000 years ago. The sport was officially added to the 2000 Olympic Games and has gained rising interest over the years. Due to factors such as competition rules and scoring structures from the World Taekwondo Federation (WTF) 2014 Rules and Interpretation [3], there is a much greater focus on kicking techniques over punching techniques. Punches are awarded with 1 point whereas different types of kicks can earn up to 4 points. Nevertheless, punching is still a very effective aspect in the practice of the art, and often used in close distance self defence. Taekwondo studies have also shown clear health and strength benefits among young [4] and senior citizens [5]. Both studies demonstrated the improvements in balance through practising Taekwondo. By understanding the Taekwondo punch techniques through vision and inertial sensors, we aim to present data-based insight into the punching kinematics used in the competitive global art of Taekwondo.

The purpose of this research is to use vision and inertial based sensing analysis to provide insight into the kinematics of martial arts punching techniques for the purposes of enhanced training through system feedback. Sensor-based coaching for sports has been a rising area of focus in recent years and the use of technologies in sports has become more widespread [6,7,8]. Accelerometers are also commonly used to observe properties of physical activity and provide important information about joint and body orientations [9]. Some martial arts are hundreds and even thousands of years old, yet our understanding of them is being augmented by the capabilities of technology [10,11]. They may also augment our understanding of physics, such as the research in [12], which uses Aikido as a visual education tool for teaching the science. We mainly focus on two main Taekwondo punches, the sine-wave punch and the reverse step punch. Extensively, we conduct further experiments to compare the kinematics of the Taekwondo punches to those of Hapkido and Shaolin Wushu which have different punching philosophies. We invited three high ranking black belt practitioners of each style to demonstrate the feasibility of our system and provide insight into the unique properties of each punching style. Upon analyzing each set of data, we provide system guided recommendations based on the experiments to maximise the energy flow and the impact forces.

Energy flow in martial arts refers to the changes in kinetic and potential energy within a technique. The energy build-up leading to a technique is affected by the prior motions such as the steps, stance, rotation and joint torques of the practitioner. Reference [13] emphasizes that most of a martial arts technique’s power is derived from the hips prior to the execution of a punch and details the energy transfer through performing hip snaps. In a sine-wave motion, the practitioner moves down-up-down from the halfway neutral position. When the practitioner moves down into a technique, they are converting gravitational potential energy from their centre of mass into kinetic energy [14]. The purpose of this is to build kinetic energy from the body’s motion to transfer more energy into the punch at the moment of impact.

This research combines aspects of human biomechanics, dynamics analysis and martial arts theory to present a novel analysis of human motion through high resolution sensing. In doing so we aim to further the understanding of human movement and kinematics by analysing the capabilities of the human body through the art of Taekwondo. In particular we focus on optimising the energy flow of the motion to increase impact force of each punch. We do this through recording data of the impact forces, velocity, position, acceleration and momentum of each punch and analyzing the kinematics in line with vision data provided by high resolution cameras.

Taekwondo was developed in the 1940’s by a combination of Korean masters from a number of martial arts background including Okinawan karate, Chinese martial arts, and the ancient Korean traditions Taekkyeon and Gwonbeop [15]. Taekwondo originally called Tang Soo Do was practiced by South Korean soldiers in Seoul in the 1950’s. It was in the 1960s that the art was separated into the WTF and the International Taekwondo Federation (ITF), due to disagreements among high ranked generals about the art [16]. The WTF and the ITF are the major styles in modern Taekwondo. Although both are a form of Taekwondo these two factions are vastly different, each having their own forms, sparring system and style of movement. The leader for the WTF is Dr. Kim Un-Yong and the leader for the ITF is General Choi Hong Hi, who is mostly known for his proposition of the Theory of Power [17].

Several methods have been investigated for analyzing human motion in the field of martial arts. Motion-based analysis in martial arts has widely been used for optimising techniques [18], while interactive systems are used for teaching beginners [19]. Alternatively, technology in martial arts is also used for scoring, such as the system presented in [20] which uses machine learning to recognize and score Taekwondo Poomsae hand motions. A similar approach uses inertial measurements units for performance analysis [21]. The research in [22] presents a mixed reality that uses a RGB camera and head-mounted display system for real-time 3D human pose estimation and guidance. It uses a learning network and optical flow to guide a student to the correct position. This is in line with our objectives to develop a system for computer aided training and analysis. Research presented in [23,24] highlights the use of motion capture systems to analyze 3D human pose and its benefits in augmenting martial arts training. Extensively, vision based sensing systems have been used in other areas of sporting such as measuring kinematic properties of weightlifters and identifying fine differences in their snatch techniques [25]. The research in [26] developed a system called SensorHogu that consists of multiple wearable piezo-electric sensors to measure impact forces on Taekwondo athletes. This is designed to assist judges in determining the contact and force of strikes in competition. In the work presented by [27], wearable gyroscopes and accelerometers are used to measure the hand movements in Wing Chun. A study in [28] measured Taekwondo punching forces by embedding an accelerometer and gyroscope in a punching bag. The study showed promising results however it did not analyze the Taekwondo reverse step punch and sine-wave punch. It also did not include vision based analysis which is important in breaking down the finer dynamic details of the technique. We have included these components in our system for additional sensor-based perspectives on more Taekwondo techniques.

Further martial arts research using sensors [29] considered the force differences between a straight punch, elbow and palm strike in the ground and pound position. Using a force plate they were able to determine that palm strikes produced the greatest force among male and female practitioners.

Through the advancements of technology we are gaining more insight into martial arts biomechanics than ever before. This insight is useful as it allows us to understand the components of each technique in order to better develop it and in turn, teach it to another. We have previously conducted research to analyze human posture through embedded sensors and coaching feedback [30]. Although in our previous research we used force-sensitive resistors, this is not so suitable for high-speed applications such as martial arts, which is why we have chosen to use vision and inertial sensors for this study. The study presented in [31] utilized a full body IMU suit to compare the boxing punch techniques between senior and junior groups. The results revealed that those in the junior group used their shoulder more in the contribution to each punch. We have previously used sensors embedded in clothing to track human motion in real-time and control a robotic arm [32]. Although this was not directly related to martial arts, it did provide us with a foundation for understanding the use of sensors to track different joints and muscles of the human body. Sensor-based dynamic analysis in Taekwondo has mainly focused on kicking biomechanics and motion, such as the research presented by [33] which targeted athlete endurance during kicking. The research in [34] used 2 high-speed video cameras to analyze the kinematics of the different phases in a Taekwondo sidekick. The research presented by [35] analyzed the components of the Taekwondo roundhouse kick using a motion reconstruction system and EMG (electromyography) sensors. The results revealed that the knee and hip extension in the loading phase of the kick, were crucial components in the execution of the roundhouse kick on impact. Sensor-based dynamics studies have also been conducted to compare the kicking differences in Taekwondo between WTF and ITF [36].

While there have been various studies focusing on kicking biomechanics in Taekwondo, our focus emphasizes the sensor-based dynamics analysis of punching techniques and the motion differences between martial arts styles. Furthermore, most systems have opted to use either a vision based system or an inertial based system worn by the user. Our system combines both vision and inertial components to provide a more in-depth insight into the biomechanics of Taekwondo. To our knowledge, there is no available non-invasive system for Taekwondo punching measurement that has a simple setup and can be easily reproduced. Our system was designed with the intention for non-invasive (non-wearable) measurement for Taekwondo punching techniques. It can easily be reproduced with a standard punching bag, off the shelf accelerometers and consumer accessible cameras. Many state of the art systems require complex setups [28], are invasive on the practitioner [26] or focus primarily on the more popularized Taekwondo kicking techniques [35].

All martial arts styles have a methodology behind each technique in their style. Something as simple as an optimal punch is heavily contested between styles due to the number of variables in human motion involved in executing a punch. This consists of fist kinematics, arm motion, trunk rotation and lower limb movements [37,38]. A common straight punch between styles, involves extending the arm outwards and impacting the opponent directly with the fist. However, there are even differences in the forward kinematics involved in this simple punch. Styles argue between fist rotation, orientation and arm extension upon impact. The straight punch technique is also modified in Taekwondo as the reverse-step punch by punching with the rear hand instead of the front hand [39]. More complex theories such as the Taekwondo sine-wave punch [17] even consist of complex body motions of shifting your weight in a sine-like motion.

It is clear that each punching technique within Taekwondo alone contains specific biomechanics and motion properties in its execution. When considering the world of martial arts and all the various styles, the sheer scale of dynamics measurement we can gain through sensors becomes a key area of scientific interest. Our combination of vision and embedded sensing aims to shine light on the theoretical explanations of the Taekwondo reverse-step punch and sine-wave punch. In-depth kinematic comparison between Taekwondo, Shaolin Wushu and Hapkido further highlights differences between the punching dynamics and biomechanical properties.

Our key objectives (O) in this research include:

- O1: Develop a sensor-based understanding of martial arts punching dynamics, in particular Taekwondo, to provide deeper biomechanics insight for practitioners;
- O2: Develop a platform for analyzing various martial arts techniques with high resolution accuracy;
- O3: Analyse the kinematics and motion of the Taekwondo techniques in a lab testing environment using a combination of inertial and vision sensing methods;
- O4: Investigate different methods to accurately measure and record the velocity and force exerted by the practitioner;
- O5: Compare and evaluate different punching techniques from various martial arts styles to demonstrate the feasibility of our system and highlight unique properties in each style; and
- O6: Conduct experiments with high ranking black belt practitioners.

As a first step in our research, we conducted an in-depth review of Taekwondo punching techniques and punching biomechanics of other common martial arts styles. Understanding the dynamics involved provided a guideline for our observations and system setup.

## 2. Materials and Methods

### 2.1. Human Biomechanics in Taekwondo

In this section we present an overview of the Taekwondo punches analyzed in this study. The punches are the straight punch (for reference), reverse-step punch and sine-wave punch of Taekwondo. These are the main punches analyzed in our research and compared to techniques from other martial arts in our follow-up experiments. Certain figures presented in this section are extracts from our experiments and used as a guide for explanation.

The straight punch is the primary Taekwondo punch as it allows the practitioner to make contact with the target in the most simple and practical way [40]. The strike is driven forward directly to the target in a linear fashion and the motion of the punch is propelled prominently by the shoulder and triceps muscles. In order to deliver a more powerful punch, the practitioner must engage other muscles throughout the whole body, such as the trunk, legs and hips.

Ideally upon impact the striking arm needs to remain slightly bent as the force of the impact could cause the elbow to hyper-extend which may be harmful to the practitioner. Therefore, the key to a powerful Taekwondo punch is the practitioner making contact with the target before their arm has been fully extended. This allows the practitioner to drive the remaining motion of the punch through the target thus delivering a greater momentum across to the target.

**Figure 1:**
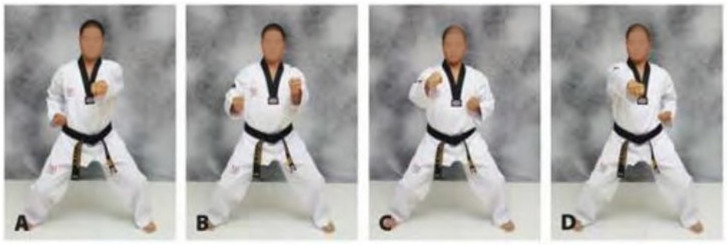
Taekwondo straight punch in a horse riding stance, (**A**) Straight punch with left hand in horse stance, (**B**) Rotating left hand to vertical position, (**C**) Retracting left hand and launching straight punch with right hand simultaneously, (**D**) Fully extended straight punch with right hand [39].

Figure 1 shows a practitioner executing a straight punch in conjunction with the horse riding stance also known as the sitting stance [39]. Park states that the horse stance for punching is not used in combat or sparring but rather for training purposes due to the disadvantage of the practitioner’s frontal area being overly exposed [39]. He states further that this style is widely used in training exercises to help develop punching techniques as well as leg muscle strength.

We describe the reverse-step punch and sine-wave punch in greater detail as they are more unique to Taekwondo and the motions involved are more complex than the straight punch.

**Figure 2:**
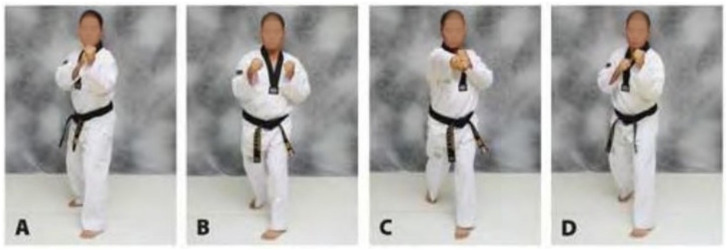
The Taekwondo reverse-step punch (**A**) Fighting stance, (**B**) Launching punch with rear hand while rotating the hip, (**C**) Fully extended punch with rear hand, (**D**) Retracting striking hand back to fighting stance [39].

The reverse-step punch, as depicted in Figure 2, is perhaps the most powerful strike in the Taekwondo arsenal of offensive attacks, the reverse-step punch is also a linear driven strike. It is delivered with the rear hand from a guard stance fighting position. Park states that the power of the punch comes from engaging the hip muscles to allow the upper torso to create torque to further drive the punch into the target [39]. The power and speed generated from this strike makes it a preferable technique to use in competitive Taekwondo sparring. Essentially the reverse-step punch is a straight punch delivered with the rear arm. The steps are depicted in Figure 2. The strike starts with the practitioner in the normal guard position, the hips and shoulder then twist forward to face towards the target then the rear striking arm is snapped out rapidly and extended straight into the target. The reverse step punch is depicted in greater detail in our results.

**Figure 3:**
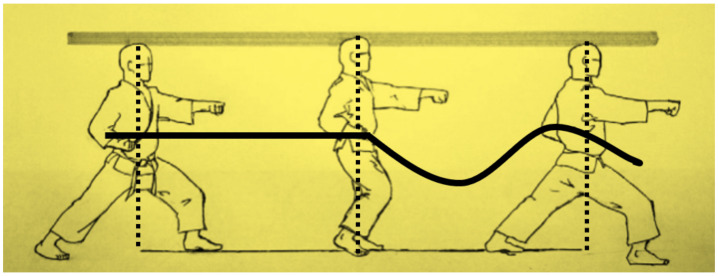
The Taekwondo sine-wave punch—image based on article in [41].

The sine-wave punch was founded by General Choi in 1983 [17] He emphasises lifting the body mass, thereby increasing potential energy and then converting this stored energy into kinetic energy by dropping the body mass into the technique. This can be seen in the Figure 3. The sine-wave punch is performed with a smooth motion. General Choi states that the practitioner must raise their body slightly when moving forward and drop as they step into the stance. Furthermore, in his works [17] General Choi states that the formula used to calculate the power of any punch is given by Equation (1) where *m* is the applied mass (body-weight) behind the punch on impact and *v* is the velocity of punch on impact.

(1) $$P=\frac{1}{2}m\times v^{2}$$

This is representative of kinetic energy, where the punch force is a result of the mass and velocity squared of the impacting object. Greater kinetic energy or force is obtained from maximizing the mass (applied body weight behind a punch) and speed. By moving in a sine like motion before executing the punch, the practitioner can improve their punching power by generating more velocity and increasing applied body weight through changes in kinetic energy.

We examine in detail the motions and kinematics involved in the reverse-step punch and sine-wave punch in our results and discussion of this paper. We use the theories for each technique presented in this section as a guide for key observations in our research.

### 2.2. Kinematics between Martial Arts Styles

In Grenville Harrop’s [42] he describes the experimentation conducted by National Geographic’s Fight Science program [43]. Four fighters from various different martial arts backgrounds including Boxing, Taekwondo, Karate and Kung Fu were required to perform their strongest punch. The results are presented in Table 1. The results are considered to be representative of the fighter not the style. It was clear through the fight science video that the fighters’ weights were different. In-line with the kinematics equations we previously presented, this greatly affected the final result as weight is a major factor in the power output of a strike. The weights of the fighters were not published but it should be noted that the Wushu Kung Fu expert was visibly much lighter than the other participants. Table 1 shows that the Boxer had the highest punching force, measured at 4417 N. The lowest measured punching force was 2722 N by the Kung Fu master, although his fist speed was considered to be extremely fast. Keeping the difference in weight of the fighters in mind, the results indicated that weight and technique greatly affected the punching force. This presents a contradiction with common martial arts theories that state that the major factor in creating power is speed. Based on these findings, we considered the force to weight ratio and effective body mass during our experiments. These are important factors in evaluating the effectiveness and kinematics of individual techniques across practitioners.

To appreciate the forces from these fighters the physical damage from these forces must be defined. In an article by Choi [44], biomedical engineer Cindy Bir found that there is a 25% chance that an average person’s ribs would crack taking a punch force of 3300 N. Furthermore, it was stated that a punch delivered at 4000 N could fracture the femur would have a high chance of fracturing the femur.

### 2.3. Vision Sensing System Implementation

Video motion analysis is a method used to analyse moving objects, using sophisticated vector mapping software to obtain the acceleration, velocity and displacement of the object. This is a popular method in sports analysis and coaching. The ability to analyse each video frame allows coaches to break down the dynamics of the technique executed and identify areas to be improved upon. In the case of the Taekwondo sine-wave punch, the software is able to analyse the displacement, velocity and acceleration specifically of the fist and the punching bag. We used this to template match with the theoretical explanation of the sine-wave punch and analyze the dynamics involved.

**Figure 4:**
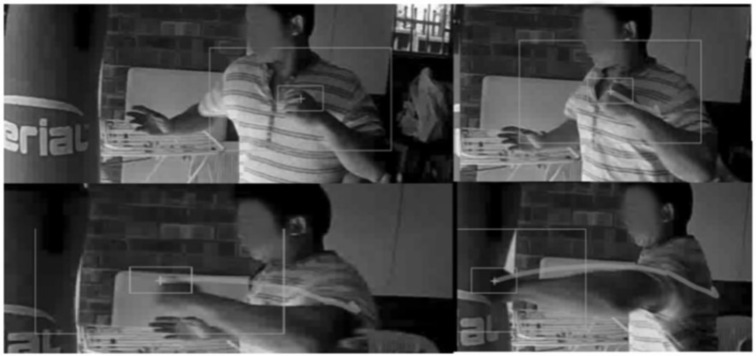
Kinovea motion tracking example [45].

We used the motion analysis software Kinovea [45] to analyze the data from two high speed cameras. We used one camera focused on the practitioner and another camera focused on the motion of the punching bag. A 100 mm marker was drawn on the front of the punching bag and used to calibrate the frame using the Kinovea software. Once calibrated the motion of the punch could be tracked using a tool which automatically followed a selected point on the video. If the tracking was misaligned the user could manually drag the tracking point to the desired position on the video and individual frames. Dividing the distance by the time produced the velocity of the motion. The higher the frame rate the more intervals at which the velocity could be measured which gave a more specific result to the executed motion. Once the motion had been tracked through each frame, the software automatically calculated the velocity of the motion through each frame by analysing the distance vector tracked over the time elapsed. For this experiment we used the GoPro Hero 3 [46] which allowed us to record video at 720P resolution (720 lines each 1280 pixels wide) and 120 frames-per-second (FPS). An example of the motion tracking provided by the Kinovea software is depicted in Figure 4.

In order for the motion analysis software to work we ensured the video recording data was taken perpendicular to the motion of the punch. This allowed the software to sample the motion in the X-Y plane. If the video camera was placed directly in front of the practitioner, the software would not be able to analyse the data correctly as the software relied on measuring the change of distance over a certain amount of time. The same was the case for video cameras set up isometric or angled to the scene. Furthermore, the distance could not be automatically measured using the software unless it had been calibrated to the real world scale using a calibration marker. Usually a calibration marker is an object with known length, for example the 100 mm marker we have used equates to 65 pixels in the video based on our setup. This ratio will vary depending on the distance of the camera taken relative to the plane of motion. We ensured that the marker was on the same plane of the punch performed so that the the analysis would be in the correct perspective view. We further prepared our system by taking real-world measurements of the practitioners’ heights, punching bag dimensions and marker sizes. Once the vision based system was completed and setup correctly, we began implementing the inertial sensing system.

### 2.4. Inertial Sensing System Implementation

We used an accelerometer in this project to measure the instantaneous acceleration to determine velocity and displacement of the punching bag. Instantaneous acceleration is the limit of the rate of average acceleration as the time interval approaches zero. This is also known as the derivative of velocity. We used the common kinematic equations of acceleration for analysis of the punching bag’s motion.

The accelerometer used was the ICSensor Model 3140. It has a range of ±50 g which had enough resolution for our research purposes. The sensitivity of the accelerometer was set to 40 mV/g. This is the calibration setup that allows the recorded data to be interpreted into accurate force measurements for our application.

One of the many static errors of the accelerometer we encountered was bias. This occurs when the accelerometer is out-putting shifting values even when there are no forces acting on the device. This may have been due to the build quality of the accelerometer where the proof mass may have a null position that it offset from the assumed null position. We handled this error by subtracting the bias error from the output for a true acceleration. Misalignment between the accelerometer and the body of interest can also cause an error on the output. For example, if the body of interest is moving along the x-axis but the accelerometer is slightly angled from the body then the output of the accelerometer will be a combination of both the X and Y plane. However, although bias was an occurrence, it did not significantly hinder our observations as we were able to determine relative kinematics between punches and practitioners with enough data insight when coupled with the vision sensing system. Our measurements also occurred over very brief intervals of time.

### 2.5. Measuring Martial Arts Techniques

Using the inertial sensing system, we calculated the force exerted from analysing the momentum of the punch and the bag. This is also known as linear or transitional momentum and is the product of the mass and the velocity of an object. In our application for this research the punching bag will have a mass and the practitioner’s fist is the force that drives the bag in a forward motion at some velocity. Therefore the momentum of the bag can be calculated using Equation (2).

(2) p=m×v,

where *p* is momentum, *m* is the mass of the bag and *v* is the velocity of the bag.

Impulse force was previously used as an injury criterion [47,48]. We considered here the impulse (*I*) in each motion by multiplying the force (*F*) of the fist by the impact time (ΔT) using Equation (3).

(3) I=F×ΔT.

In our inertial system we also considered the conservation of momentum in dynamics measurement. The law of conservation of momentum is for a collision occurring between object 1 and object 2 in an isolated system. The total momentum of the two objects before the collision is equal to the total momentum of the two objects after the collision. That is, the momentum lost by object 1 is equal to the momentum gained by object 2. The logic behind this is when 2 objects collide the forces between the two objects are equal but opposite in direction (Newton’s Third law). It is said that the two objects will collide together for an equal amount of time. This is considered as ‘impulse’, since the objects experience equal and opposite impulse the formula can be written as Equation (4).

(4) $$m_{1}\Delta v_{1}=-m_{2}\Delta v_{2}.$$

Using Equation (4) in our system, $m_{1}$ is the mass of the punching bag, $\Delta v_{1}$ is the difference between the initial and final velocity of the bag, $m_{2}$ is the effective mass which the practitioner puts behind the punch and $\Delta v_{2}$ is the difference between the initial and final velocity of the practitioner’s fist.

This completes the explanation of our sensing system used for analysis. In our experiments we combined both the vision and inertial sensing systems and compared the data to make observations. Once the complete sensing system was implemented and setup, we proceeded to conduct experiments to analyze the dynamics involved in the key martial arts styles and techniques of Taekwondo, Shaolin Wushu and Hapkido.

## 3. Results

We first described our experimental setup in relation to our developed system. We then proceeded to use this setup to conduct a system feasibility test followed by several experiments analyzing the punching dynamics of Taekwondo. Finally, we compared the results of Taekwondo punching dynamics with punching kinematics of the martial arts styles of Hapkido and Shaolin Wushu.

### 3.1. Experimental Setup

We firstly connected our accelerometer to an oscilloscope to display and record the readings. The oscilloscope was set with a trigger point so that minor movement and vibration did not set off the recording mode. The accelerometer was placed directly behind the punching bag where the strike would hit to maximise the reading and increase consistency in the results. As the strike impacts the bag it causes the bag to accelerate forward, which causes the accelerometer to output a voltage.This voltage is then converted to measurements of gravity (g) through dividing by the accelerometer’s calibration data. This is how we computed impact force and the other resultant properties of the punch. The punching bag used weighed 52 kg.

**Figure 5:**
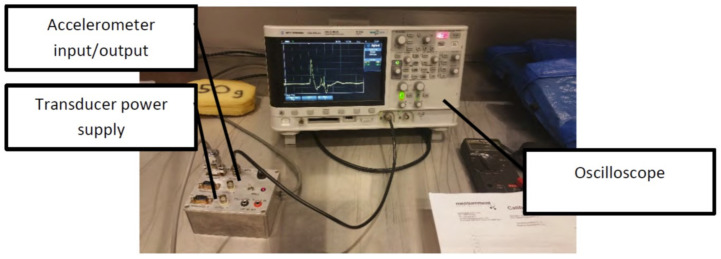
Inertial measurement setup shows the accelerometer output to the oscilloscope through the transducer power supply.

The oscilloscope was set to record every 10 ms at 500 mV per division. The oscilloscope setup is depicted in Figure 5. It was important to set the oscilloscope at 500 mV per division as setting it too low may cause the recorded data to cut the peaks off the result. Our pilot trials showed that for a regular punch 500 mV was sufficient for recording the data. For other high force techniques such as kicks, this setting should be re-adjusted to suit the setup.

The impulse and momentum measurement setup consisted of two high speed cameras, one recording the bag’s movement and the other recording the practitioner’s movement. The cameras were placed perpendicular to the plane of the practitioner striking. Marker points were placed on the punching bag to allow a single point to be tracked on the Kinovea program. The main data obtained from the camera recording the practitioner were the fist velocity upon impact and the change in height as the practitioner performed their technique. The data obtained from the camera recording the bag was the velocity at which the bag travels whilst being struck. A calibration object was placed on the plane of motion so that the video pixels could be calibrated to the real world dimensions. In this setup a 100 mm ruler was placed in the striking zone for camera used on the bag.

The practitioner’s height was also used to calibrate the camera and additionally used to track their motion. Initially using the 100 mm ruler on the camera, tracking the practitioner’s motion was proven to be inaccurate as the margin for error from scaling a 100 mm object to the practitioner’s height was too high. Using the practitioner’s height to calibrate was a better option as the scaling error was dramatically reduced.

**Figure 6:**
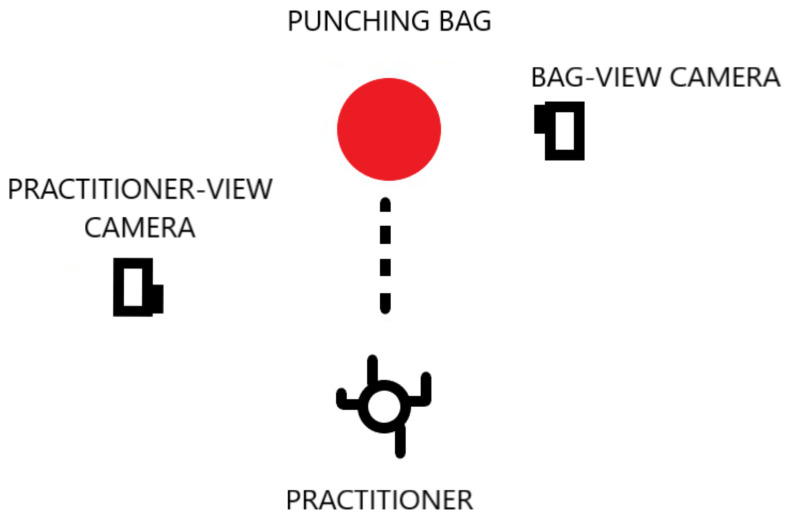
Vision sensing system camera experiment setup.

The video camera used was GoPro Hero 3 and the video was set up to record at 720P at 120 FPS. The camera setup for the experiments is depicted in Figure 6. This method of obtaining data was still prone to minor errors as there were some factors that would cause an error in the readings such as calibration error and tracking error. However, the results gave a close enough indication of the velocities in order to perform the calculations for the purpose of this experimentation.

### 3.2. System Feasibility Experiment

**Figure 7:**
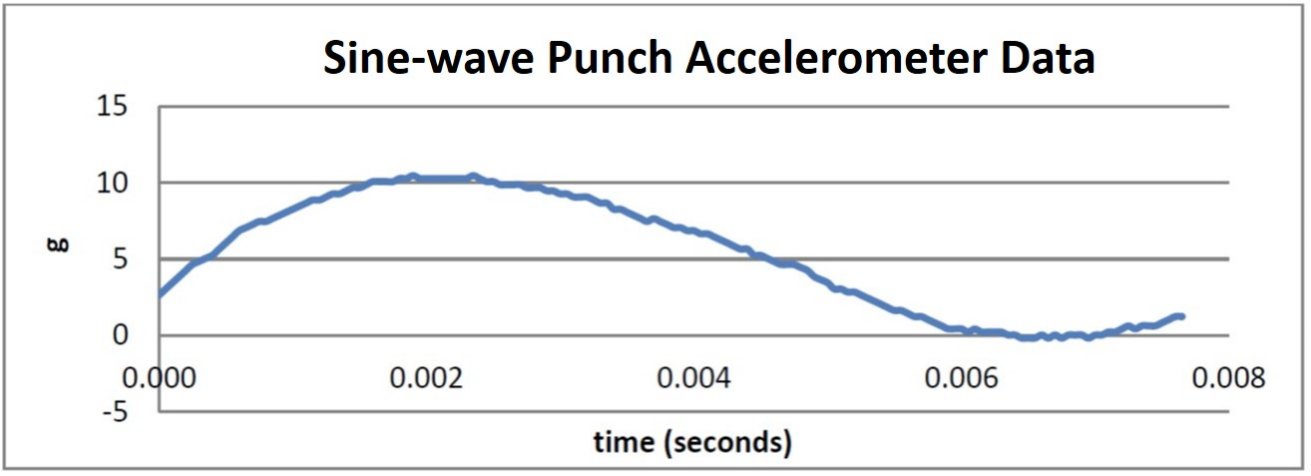
Sine-wave punch accelerometer data.

**Figure 8:**
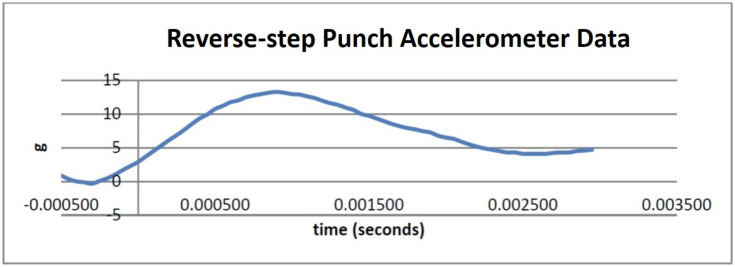
Reverse step punch accelerometer data.

In this experiment, we tested the feasibility of our sensors-based measurement system by evaluating the impact force of the sine-wave and reverse-step punches. We used both results from our vision and inertial system in evaluating our system feasibility. Impact force was defined as a high force or shock applied in a very short amount of time. During a perfectly inelastic collision, the object that has been struck will deform and this deformation will absorb a portion of the force applied. However, high velocity collision does not provide enough time for this deformation to occur. Thus, in our observations, the bag behaved in a different way. It becomes stiffer and most of the forces travelled through the medium as vibration. This was evident through the recorded graph from the accelerometer placed on the punching bag as seen in Figure 7 and Figure 8.

We asked the Taekwondo practitioner to perform 3 instances of the sine-wave punch and 3 instances of the reverse-step punch. The data obtained from the accelerometer was analysed and the results are displayed in Figure 7 and Figure 8. Using the Kinovea software and our system setup, we investigated the changes in height of the practitioner given by the vision sensors and combined these data with the impact force readings produced by the accelerometer on the punching bag.

### 3.3. Taekwondo Punching Biomechanics Experiment

After demonstrating the capabilities of our sensing system to analyze martial arts techniques, we proceeded to examine the Taekwondo punches in more detail. In order to analyze the Taekwondo techniques with our system we asked a 6th Dan Black Belt Taekwondo Master to participate in the study. For the first set of experiments, we asked the Taekwondo Master to perform the sine-wave punch and reverse-step punch 3 times. The repetition of the technique was kept at 3 to reduce fatigue and technique loss from over-performance. The data were recorded by the accelerometer fitted on the punching bag and our dual high-speed camera setup. Afterwards, we analyzed the data using the Kinovea software paired with our video coded observations. The velocity results are presented in Table 2 and the impulse measurements are presented in Table 3.

**Figure 9:**
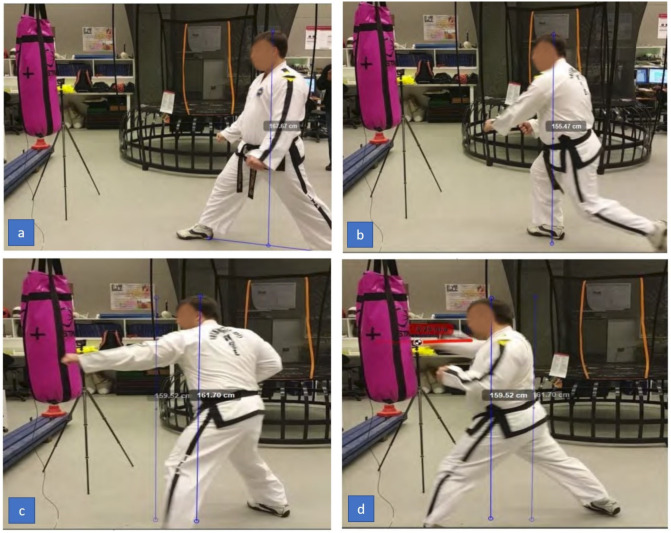
Taekwondo reverse-step punch motion (**a**) preparation phase (**b**) forward step (**c**) retracting punching hand (**d**) striking with punching hand and drawing opposite hand back.

**Figure 10:**
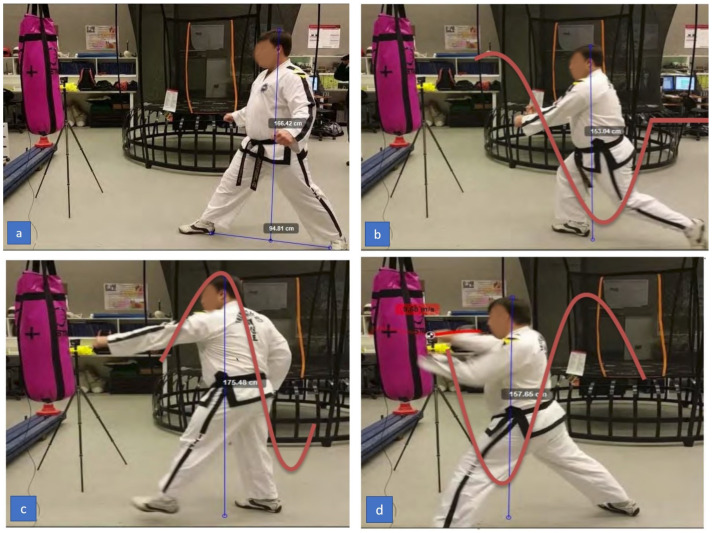
Taekwondo sine-wave punch motion (**a**) preparation phase (**b**) forward step and lowering height (**c**) pulling back the striking hand and increasing height (**d**) extending punching hand and dropping height simultaneously.

A frame-by-frame recording of the Taekwondo practitioner’s reverse-step punch is shown in Figure 9 and the sine-wave punch is shown in Figure 10. The Kinovea software assists in labelling the height and real-world measurements in each frame.

The reverse-step punch motion analyzed by our system is broken down based on key frames. We list these items and refer to Figure 9 as taken by our vision sensors.

(a) The practitioner is in a wide stance starting position. His body center line is lined up to the target. His starting height is approximately 1670 mm from the ground. He adopts the same starting position as the sine-wave punch.
(b) The practitioner takes a step forward and lowers his stance to 1550 mm. At this point most of the weight is channelled to the right leg ready to spring forward.
(c) The practitioner draws the right arm back getting ready to execute the punch, steps forward and elevates his height to 1610 mm.
(d) The practitioner extends his punching arm for the strike. Just before the impact the fist velocity peaks at 9.29 m/s. We notice the left arm has been drawn back as compared to the sine-wave punch where it was extended. The act of pulling the arm back engages the core muscles which rotates the body and generates power into the punch. The height change is linear as compared to the sine-wave punch. There was only a difference of approximately 25 mm between the highest and lowest points.

The sine-wave punch motion analyzed by our system was broken down based on key frames. We list these items and refer to Figure 10 as taken by our vision sensors.

(a) The practitioner begins in his wide stance starting position. His body center-line is lined up to the target. His starting height is approximately 1660 mm from the ground;
(b) The practitioner takes a step forward and lowers his stance to 1530 mm. At this point most of his weight is channelled to his right leg ready to spring upwards. The practitioner’s body height follows the sine wave curve;
(c) The practitioner draws his right arm back getting ready to execute his punch, his stance is at its highest peak at approximately 1750 mm. At this point he has gained the most potential energy during the motion; and
(d) The practitioner extends his punching arm for the strike. Just before the impact, the fist velocity is max at 9.68 m/s. We observe his left arm is still extended, this keeps his body centred to the target allowing him to direct his line of attack in a forward motion. His height is at the lowest at 1570 mm. At this point most of the potential energy has been converted to kinetic energy.

We then calculate the effective body mass using the conservation of momentum. The body effective mass is the mass that the practitioner is able to put into the strike. On this particular set the results do not reflect the actual effective mass of the strike as the deformation of the bag causes only a portion of the bag to accelerate. Therefore the effective mass of the bag that has been accelerated is smaller than the actual weight of the punching bag (52 kg). However, the results are relative to each other and therefore can be used to compare results within this experiment. The effective body mass for each trial is also presented in Table 3.

### 3.4. Punching Kinematics Comparison Experiment

**Figure 11:**
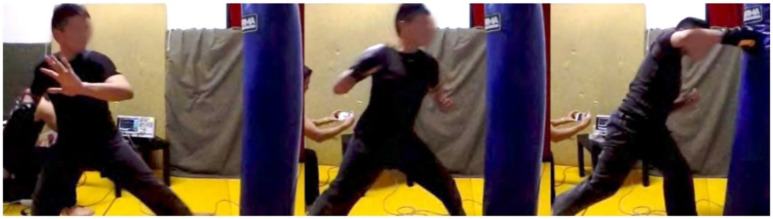
Hapkido right-cross punch motion.

**Figure 12:**
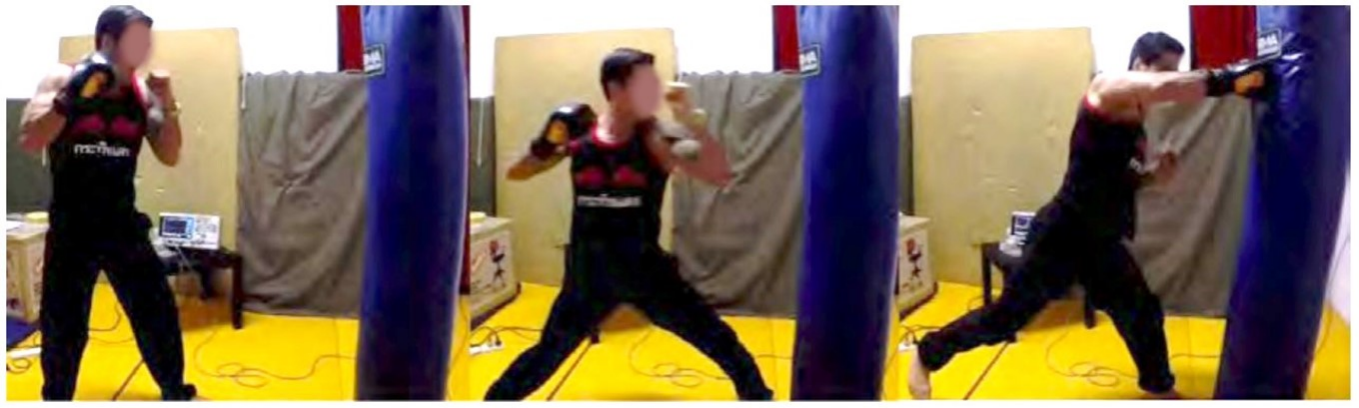
Shaolin Wushu right-cross motion.

After evaluating the key Taekwondo punching techniques, we conducted an experiment with two other practitioners from different martial arts backgrounds to compare the differences in punching motions and kinematics to the Taekwondo results from our first set of experiments. We invited a black belt in Hapkido and a Master in Shaolin Wushu. Both practitioners had over 10 years experience in their respective martial arts styles. They both performed a right-cross punch from a stand still position for 2 trials. The right-cross punch was agreed to be a strong punch in both styles and a good baseline for comparison to the Taekwondo punches and between all 3 styles under analysis. Similar to a boxing right-cross they both generate power through the rotation of the body and driving the power from the legs into the punches. Figure 11 and Figure 12 depict the motion breakdown of each practitioner while performing the right-cross punch. The impact force of each punch is outlined in Table 4. We combined these with the results from the main Taekwondo experiment and present a full comparison in Table 5.

As captured by our vision sensors system, from Figure 11 we can observe the starting stance of the Hapkido practitioner is low with his back knee bent. Most of his weight is on his back foot. As he rotates his body the weight and center of gravity starts to shift forward as shown by the image in the middle of Figure 11. He finishes his strike with all his weight on the front foot, we also observe that his rear leg is off the ground.

As captured by our vision sensors system, from Figure 12, the Wushu practitioner starts with the conventional boxing stance and then extends his left leg forward lowering his center of gravity, allowing him to drive his punch upwards starting from his rear leg. Similar to the Hapkido practitioner’s punch they both lower their stance and drive upwards in a forward motion to deliver the strike. We can easily observe that both practitioners’ rear legs end up in the air as all their weight gets shifted to the front foot.

## 4. Discussion

### 4.1. Evaluating System Objectives

In this study we outlined 6 main objectives O1–O6 in our Introduction section for research and evaluation. These aims are addressed individually in our discussion and evaluated in terms of the results produced with O1 being the major design objective for this research.

### 4.2. System Feasibility as a Platform for Martial Arts Dynamics Analysis

**Figure 13:**
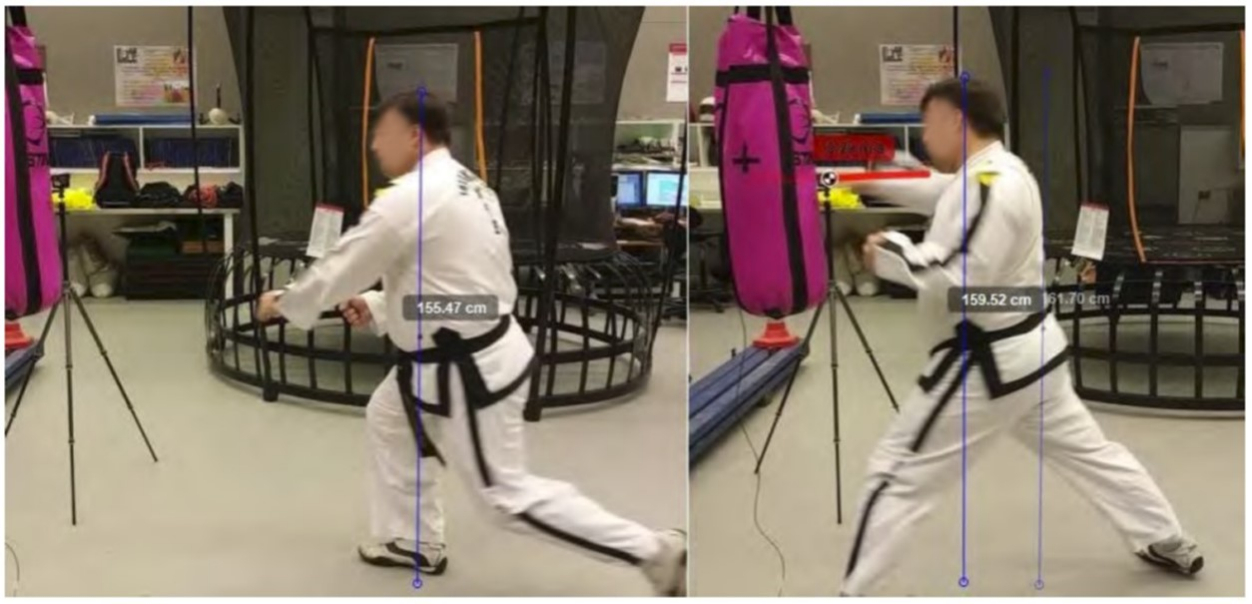
Reverse-step punch observed height differences during motion.

**Figure 14:**
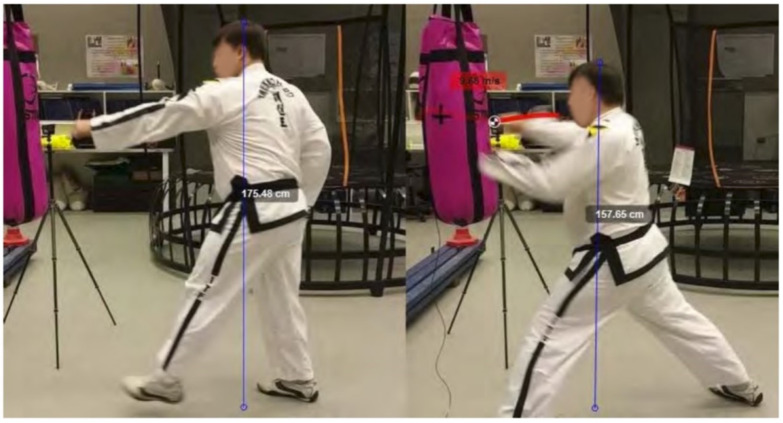
Sine-wave punch observed height differences during motion.

Firstly, to evaluate objectives O2 and partially O4 we considered the feasibility of the sensing system in measuring impact force and its capabilities in visualizing changes in motion during the techniques. The camera and tracking results shown in Figure 13 and Figure 14 accurately reflect the true height values of the practitioner during execution of the motion. Furthermore, the graphs displayed in Figure 7 and Figure 8 show the acceleration values for each punch as measured by the accelerometer on the punching bag and reflect the motion of the bag as recorded by the cameras.

As seen from Figure 8 the acceleration measured from the sine-wave punch was approximately 10 g. The calculated momentum was 45.24 kg·m/s from our video analysis. Notice also that at a lower impact, the time interval dragged out to 0.006 seconds. Furthermore, from Figure 8 the impact from the reverse-step punch recorded a reading of approximately 13 g. The momentum from this punch was calculated to be 26.52 kg·m/s. Notice that for a higher impact punch the momentum was much less. The time interval was approximately 0.0025 s. By analysing Figure 8 we can also see that the impact force can be applied to our study to compare the difference in the forces of the punches.

As seen from Figure 13 and Figure 14 the two main differences between the sine-wave punch and the reverse-step punch was the change in potential energy. Potential energy is defined as the stored energy from the position it is placed relative to the ground. The higher the object the more potential energy it possesses. The formula for potential energy is given in Equation (5) where *m* is mass, *g* is gravity and *h* is height.

(5) PE=m×g×h.

On the other hand, kinetic energy is the result of motion. If the object is dropped from a height it will gain velocity. We have previously outlined the formula for kinetic energy in Equation (1). The relationship between potential energy and kinetic energy can be represented by Equation (6).

(6) ΔPE=ΔKE.

Figure 14 shows that the practitioner started at a height of 1754.80 mm. At this point he had a potential energy of 2142.50 J. At the point of contact his height was 1578.60 mm. Based on these changes of height, the potential energy was calculated to be 1930.10 J and the difference in potential energy was 212.4 J. From the relationship between potential energy and kinetic energy given by Equation (6), we can then represent velocity as in Equation (7).

(7) $$v=\sqrt{\frac{KE}{0.5\times m}},$$

where KE is kinetic energy and *m* is mass. Using the definition in Equation (7) we calculated the change in potential energy of the sine-wave punch to be 1.844 m/s. The reverse-step punch in Figure 13 shows the practitioner’s peak height was 1617.00 mm whilst executing the strike. The potential energy was calculated to be 1979.67 J. At the point of contact his height was 1595.20 mm and the potential energy was 1952.98 J. The difference in potential energy was then calculated to be 26.68 J. The velocity gained from the change in potential energy was 0.653 m/s.

These results prove that our system was capable of accurately measuring the punching motion and changes in kinetic and potential energy of the practitioner. It also shows that our vision and inertial sensing systems complement each other in validating observations from the cameras and accelerometer. We discuss these results further in the next part of this section which presents more focus on the kinematics of the individual punches. As supported by our results in this study, we successfully completed O2 of this research and partially supported objective O4.

### 4.3. Kinematics of Taekwondo Punches

The Taekwondo punching experiment was designed to evaluate objective O3 of this study by using objective O6 as a prerequisite. We had already completed objective O6 by recruiting black-belt practitioners for the Taekwondo and martial arts comparison studies to ensure high quality data is obtained. The data from Table 2 shows that on average the sine-wave punch generated more velocity as compared to the reverse-step punch. On average the sine-wave punch fist had a velocity of 9.20 m/s whereas the average for the reverse-step punch was only at 8.51 m/s. This was also reflected by the punching bag’s velocity measurements. The differences in fist velocity can be attributed to the steps executed before the punch was delivered. From the results, the sine-wave motion of the body seemed to be creating a driving force of the fist forward that resulted in a higher fist velocity on impact. The reverse-step punch, although linear in motion, did not leverage the additional benefits of potential energy as the sine-wave punch. This can be attributed to the reduced fist velocity upon impact. There were other factors we considered here, such as the proficiency of the practitioner with each punch. Prior to the experiment, the practitioner had reported a balanced proficiency with each technique, however this interpretation may not be reflective of the actual physical execution.

The results in Table 3 show that, on average, the sine-wave punch generated more impulse velocity as compared to the reverse-step punch. This was due to the higher velocity of the fist impacting on the punching bag, as momentum is defined by Equation (2). The mass of the bag stays constant therefore the only variables are the fist velocity and the higher order derivatives of this velocity (acceleration, jerk and snap) [49].

As seen from Table 6 the impact and impulse forces were inversely proportional to each other. If we look at the first trial of the sine-wave punch, the impact force was 8834.2 N and the impulse force was 238.5 N, whereas in the third trial of the sine-wave punch, the impact force was 5341.96 N and the impulse was 312 N. It was clear that the higher the impact force the lower the impulse force. This was due to the shorter observed time from high velocity impact. When comparing the sine-wave punch and the reverse-step punch overall both impact and impulse forces for the sine-wave punch were greater than for the reverse-step punch. This clearly showed that the sine-wave punch was much more powerful than the reverse-step punch. On average the impact force of the sine-wave punch was 26.1% greater than the reverse-step punch. The impulse force of the sine-wave punch was 21.95% greater than the reverse-step punch. We attributed this to the velocity gained from the change in potential energy of the practitioner’s body leading up to the strike which was also observed in the System Feasibility Experiment.

The sine-wave motion concentrates on lifting and dropping the effective weight into the punch whereas the reverse-step punch concentrates on twisting of the torso to generate power into the punch. Figure 14 shows the practitioner’s left arm is still extended at the point of contact, whereas in Figure 13 the practitioner’s left arm is being retracted towards his body. This was an interesting observation between punches. After discussing with one of the black-belt practitioners with 20 years of experience, he explained that in Hapkido and Boxing, the cross punch’s power comes from retracting the left arm to rotate the torso whilst driving the weight of the body from the back leg to the front leg. The result from the combined movement generates the power behind the strike. This is aligned with the research in [50] which describes a similar kinetic chain amongst elite boxing athletes. It outlines that punching power can be generated from floor to fist. We look into this in greater detail in the next part of the discussion, but it provides interesting insight into the role of the trunk’s motion in combination with the retraction of the arm. During the experimentation with the Taekwondo Master, the elements mentioned by the Hapkido practitioner were evident in the reverse-step punch visual tracking results. The only difference was that the Taekwondo Master took a step into the punch rather than just shifting his weight from a stand still position like a typical boxing punch. Stepping forward firmly right before a punch has also been attributed as a key factor in increasing punching force [51]. Although step-through punches can increase power through the landing motion, they also require more time to execute. By stepping through while punching, the practitioner can also leverage effective mass into their extending arm. As mentioned previously, when the Taekwondo Master was performing his sine-wave punch, his left arm did not finish in the retracted position but was instead fully extended. This shows that he did not engage his torso into the rotating motion to generate power but instead relied on the spring-like motion from the legs to increase kinetic energy.

Our results clearly undermine the misconception that by increasing the velocity of the punch the energy of the punch will exponentially increase as well. The analysis we present demonstrates that the increase in velocity can be caused by the changes in potential energy. Therefore when looking at the sine-wave motion of the body in executing a punch, the only way to increase the energy generated is through:

1. Increasing the mass of the practitioner;
2. Increasing the acceleration at which the practitioner drops their weight into the strike; and
3. Increasing the distance in which the practitioner drops their height.

Through the results produced by our system and the analysis of individual Taekwondo punching properties, we have successfully completed objective O3 of this study.

### 4.4. Dynamics between Martial Arts Styles

The martial arts comparison study was designed primarily to evaluate objective O4 and O5 of our research aims. From Table 4, the Hapkido practitioner’s fist velocity upon contact with the bag was approximately 9.95 m/s whereas the Wushu practitioner’s fist velocity upon contact with the bag was approximately 8.35 m/s. This was observed through the video analysis software Kinovea. However, the data recorded on the accelerometer showed that the Wushu practitioner produced peak acceleration on the punching bag at 128 m/s^2^ and the Hapkido practitioner produced a peak acceleration of 126 m/s^2^. This showed that the Hapkido practitioner decelerated his punch upon contact whereas the Wushu practitioner maintained his acceleration through the punch. For this reason the Wushu practitioner was still able to maintain a consistently high impact force.

Table 5 shows that compared to the Taekwondo Master’s reverse-step punch, both the right cross punches from the Hapkido and Wushu practitioners produced a higher impact force on average. Similarly to the Wushu practitioner’s punch, the Taekwondo Master’s fist travelled only at 8.51 m/s but his peak acceleration recorded on the bag was 103 m/s^2^. This meant that he decelerated his punch. The Taekwondo Master’s sine-wave punch was much more powerful than both the Hapkido and Wushu practitioner’s punches. On average the Taekwondo Master produced a force of 6884 N for his sine-wave punch but on one of his punches he was able to reach 8834 N. It was clear that the sine-wave punch produced much more power than the right cross. However, it must be noted that all these practitioners had different weight and skill levels. The Taekwondo Master weighed 124.8 kg, the Hapkido practitioner weighed 85.0 kg and the Wushu practitioner weighed 76.5 kg. Although all practitioners had over 10 years experience in their respective fields, the proficiency with particular techniques such as advanced punches, might greatly vary depending on the practitioner’s training intensity and individual ability.

Based on the previous study conducted in [43] it was clear that the weight of the martial artist was a major factor in force output. We also observed lower comparative forces produced by the lighter Wushu fighter in our own study but could hypothesize that the weight and effective mass of the practitioner was a major contributing factor in this. To analyse the data in a way which considered the weight into the punch, the practitioners’ strikes were divided by their weight. This showed how much power they were able to utilise from their weight. The results are displayed in Table 5. As seen from the table the highest power to weight ratio was 82:1 which was the Wushu practitioner’s punch, followed by the Hapkido practitioner’s right cross. By having a higher power to weight ratio the Wushu practitioner was producing more force based on his body weight. This led us to believe that if his weight were also increased, his punching force would also be the highest compared to the Taekwondo and Hapkido practitioners. However, it is not always so straightforward, as the article in [50] describes the importance of the impulse-momentum relationship. Having more weight does not guarantee higher punching power, it is the velocity at which the weight moves (momentum) that is key in producing greater impulse force. The higher order derivatives of the velocity such as acceleration, jerk and snap may also play a role [49,52]. Factors such as individual experience that take advantage of acceleration, jerk and snap have may contribute to the differences in punching force between athletes [53].

Force to weight ratio from this experiment supported the possibility that the Wushu practitioner’s right cross might be more powerful if he were to weigh the same as the Taekwondo Master. However, it is only a possibility as there are many factors that can affect the punch such as the practitioner muscle mass, arm speed and training proficiency. Further research would be required with more practitioners of different weight classes and skill levels to determine how powerful their techniques are. Ultimately, we have completed our objectives O4 and O5 as we were able to compare and observe measurable differences between various martial arts styles while analyzing dynamics of each practitioner. Although we have completed this objectives in our study there is great potential for future works and further analysis between martial arts styles and practitioner kinematics.

### 4.5. Improvements of Punching Techniques

To increase punching power and speed practitioners can increase either their mass or the velocity of the fist. We have also demonstrated that practitioners can leverage potential energy in their motion leading up to the punch. In an article by Expert Boxing [54] many of the greatest fighters have the same thing in common which is their punching speed. The list includes; Muhammad Ali, Thomas Hearns, Ray Leonard, Mike Tyson, Roy Jones, Floyd Mayweather and Manny Pacquiao. The article states that the most important factor to attaining maximum speed is muscle relaxation during punching. A common beginner error is trying to combine speed and power together in the first step of the punching motion, but that only slows down the punch and causes practitioners to load their punches. The best method among martial arts masters involves letting the arms go, keeping balance and moving together in coordination with the hand’s speed. This state of relaxed muscles during punching was also mentioned by [17], and is the principle behind generating a smooth sine-wave motion.

The article [50] shows punching power can be increased through rate of force development, particularly stating that the goal of the athlete should be to produce very large force in a short amount of time. The article also shows the kinetic chain in boxing, where power is generated from the floor to the fist, through power being transferred to the upper body via hip and trunk rotation. Through our studies we also observed similar theories, especially when considering the Hapkido and Wushu practitioner’s right cross punches. Both practitioners generated punching power through hip and trunk rotation. The sine-wave movement of the Taekwondo practitioner focused more on converting gravitational potential energy into kinetic energy through the down-up-down movements. There were still elements of hip and trunk rotation at the moment of impact, but it was not as large as that observed of the Hapkido and Wushu practitioners.

The study presented in [55] states that the rear hand punch force can be segmented to: the arm musculature into the target, trunk rotation, and generating power from the ground using the legs. It also showed that in experienced boxers, the legs contributed to 38.6% of total punching force. This is an interesting observation as the sine-wave punch motion we observed made significant use of the legs in generating kinetic energy into a punch. The research in [51] also suggests that increasing the effective mass in a rear-hand punch is key to increasing force. In Table 3 we showed that the effective mass behind the sine-wave punch was 4.29 kg on average, which was greater than that of the reverse-step punch.

Across the martial arts there are several methods to increase punching speed and power. The first and most common is shadow boxing. This is usually done without any equipment or punching bag and involves the practitioner performing each technique in the air. This helps to focus on the technique itself as well as on balance. Another method which can be combined with shadow boxing is punching interval drills. This involves performing the technique rapidly for 15 to 20 s and resting in between. The key is the rest period which allows the muscles to regain energy to be able to punch at maximum speed again. It is a common method to build up the endurance of the practitioner. The research presented in [51,55] both suggest that punching force can be trained through exercises such as squats, vertical jumps and weightlifting variations. These exercises focus on building leg muscles and mobility, which has proven to be key in generating punching power. Finally, isometric training is often used to improve punching force. This is where force is exerted by the practitioner but the body does not move. Many may have seen this in Karate styles where the body remains still and the practitioner strikes a dummy in front of them repeatedly.

Another article by Expert Boxing [56] entitled ‘Most Important Muscles for Fighting’, emphasizes that all generated power comes from the ground. Because your legs are connected to the ground, they are most responsible for pushing off the ground to generate power throughout your body. This is also the principle behind ancient martial arts styles like Shaolin Kung Fu and traditional Karate forms. This is also the reason why horse-stance endurance training is widely taught to beginners as a first step. Proper punching is typically thrown with the legs pivoting and rotating. Fighters such as Marcos Maidana, Manny Pacquiao, Thomas Hearns, Julian Jackson, and Felix Trinidad don’t have over-developed pecs or large arm muscles but they still manage to deliver a large amount of force in their punches. All these practitioners have very defined leg muscles compared to their upper body. As future work for our research, we suggest analyzing the changes in forces under the feet and the motion of the legs while a punch is being executed. This can be done by attaching force sensors underneath the soles of the feet and attaching accelerometers to the legs of the practitioner. Using our vision based system, it would also be possible to analyze the motion of the legs, but this would be better done by a motion capture system.

From our vision sensors, we were able also verify that the more mass the practitioner has the slower the fist will travel. From a physics point of view this is correct as it would require more energy to move a heavy object as compared to a lighter object. Therefore it is recommended to build muscle mass from other parts of the body to generate power, predominantly the legs. As for the impulse force the practitioner can reduce the time of contact. By observing the impulse force formula the smaller the change in time the higher the force that was output. However, the momentum of the punch will be reduced as this is due to the limited time of contact at which the momentum can be transferred from the strike to the bag. The punch then produces more of a snap-like motion much like a boxing jab.

Through the multiple experiments conducted and results observed in our analysis, we have successfully gained deeper insight into martial arts techniques through our sensor-based system. As a result, we have successfully completed objective O1 of this study. Overall we have successfully completed all of our research objectives O1–O6. Notably, objective O5 sparks curiosity for further research insight and future works.

### 4.6. System Limitations and Sensor-Based Considerations

We have aimed to develop a sensor-based measurement system that is both simple and can be reproduced by other researchers for martial arts techniques evaluation. The data obtained from the experimentation setup may be difficult to exactly replicate as there are many factors that can alter the outcome. These factors include, for example, the type of bag used and dimensions of the bag.

The type of bag and material used to fill the bag will alter the result read by both the cameras and the accelerometer. The density of the bag defines how much the bag will deflect once it has been struck. If the bag was filled with soft material such as foam, the bag will much more easily deflect locally where the fist impacts. Another effect from soft material used is the absorbability. The material in the bag will absorb the fist upon impact. This lowers the reading from the accelerometer as the impact from the punch would have been dispersed around the bag before reaching the accelerometer located on the opposite end. A possible improvement to this system would be the inclusion of multiple accelerometers around the punching bag to gain a better overview of the readings. The study mentioned in [28] used multiple accelerometers and gyroscopes embedded in the punching bag, but this complicates the system setup and is not within our scope of developing a simple off the shelf system. In terms of materials, a medium density foam could be used that contains a good balance between comfort and absorbability. It should also be noted that some practitioners intentionally use punching bags filled with hard materials, such as stones or pebbles, to assist in conditioning their knuckles during training.

The dimension of the bag used is also a factor for system design consideration. Due to localised deflection, if the bag is too big it will bend inwards where it has been struck. This will cause a higher impulse force reading and not all of the weight from the bag will be accelerated. The effective mass is the mass which a practitioner puts behind their punch at the point of contact. In order to get an accurate reading the effective mass of the bag needs to be pre-computed but this can be rather difficult and essentially inaccurate. For measuring martial arts techniques, we suggest a standardized punching bag of given dimensions and materials be used across all scientific studies of martial arts kinematics. This would help reduce bias between studies and provide a more accurate baseline for conducting research.

The problems mentioned may be rectified by reducing the punching medium area to a minimum size as having a smaller surface area will reduce the localised deflection. The material used to fill the medium should be standardized to something denser such as sand, since there can exist many variations in foam. Instead of using an accelerometer, an alternative sensor may be a force transducer or an array of piezoelectric force sensors. By placing a force transducer on the surface where it will be struck the transducer will pick up most of the force before it is absorbed by the bag. By using a force gauge it will greatly reduce the bag deflection issue as the force is measured and converted directly from the impact. However, the position of the force gauge is crucial and will have to heavily rely on the practitioner being both accurate and precise between punches. This method has been explored in [57] where 9 flex sensors were used to measure punching force in Karate punches.

The camera used in this experiment, as mentioned earlier, was a GoPro Hero 3. The camera setting was set to record at 720P at 270 FPS. At this quality the camera was able to record the fist velocity clearly from 1 to 5 m/s. At higher speeds the image quality becomes distorted and blurry. However it gives the motion tracking software more resolution for manually adjusting the position of the punch on each frame. In order to improve the motion tracking a better camera needs to be used that can record at a higher frame rate and better picture quality. We recommend the use of high-speed cameras or action cameras as they typically are robust at breaking down fast motions into individual frames which is crucial for dynamics analysis and measurement. A potential candidate for our future experiments includes the FPS 1000. It is a small handheld camera designed to capture slow motion video at up to 18,500 FPS, and would be very useful for observing martial arts techniques in-depth.

With the suggested improvements mentioned, the experimental setup can be refined for better data outcomes. Modern digital mapping technology can also be used to model the whole body movement using methods of motion capture and embedded wearable sensing. This can generate a better view of the body flow and movement during execution of the strikes in 3D. Although we have opted for a more simple, minimally invasive and readily-available setup, there remains much to be discovered in the fields of martial arts biomechanics and an array of 3D sensors would prove to be very valuable to further this field.

## 5. Conclusions

Our analysis showed that the main difference between the 2 main Taekwondo punches was the changes of potential energy, in particular potential energy changes in the vertical height of the practitioner from the ground as the technique is being executed. From our results the sine-wave punch was observed to be more powerful than the reverse step punch. When considering the impact force the sine wave punch was 26.1% greater in force than the reverse-step punch. When considering the impulse force the sine-wave punch was 21.95% greater than the reverse-step punch. This was attributed to the velocity gained from leveraging potential energy in the steps leading up to the strike. The sine-wave motion concentrated on lifting and dropping the effective weight into the punch whereas the reverse-step punch concentrated on twisting of the torso to generate power into the punch.

Our analysis showed that the increase in velocity caused by the change in potential energy, was the main factor in increasing the punching impact force. Therefore when looking at the sine-wave motion of the body the factors that impacted the force generated were influenced by the mass of the practitioners, increasing the acceleration at which they drop their weight into the strike and increasing the vertical distance to which the practitioners drop their height.

The Taekwondo Master’s sine-wave punch was much more powerful than both the Hapkido and Wushu practitioners’ punches in the styles comparison experiment. On average the Taekwondo Master produced a force of 6884 N for his sine-wave punch but on one of his punches he was able to reach 8834 N. Force to weight ratio from this experiment supported the possibility that the Wushu practitioner’s right cross might be more powerful if he were to weigh the same as the Taekwondo Master. However, we emphasize that there are many factors that can affect the punch such as the practitioner muscle mass, arm speed and training proficiency.

In our research, we were able to complete all of our objectives outlined. Our vision and inertial sensing based system demonstrated feasibility in measuring high-speed martial arts techniques. With the use of two action cameras and a single IMU, we were able to accurately analyze the kinematic differences between martial arts techniques. This simple setup can easily be replicated by other researchers interested in analyzing the biomechanics involved in martial arts. We have developed a novel, non-invasive sensing system for measuring martial arts kinematics that can be reproduced through accessible inexpensive off the shelf components. There is further room for improvement in the system by extending the amount of inertial or force sensors used as well as increasing the number of cameras and using cameras with greater resolution and specifications.

In any martial art, there is a signature move that has been passed down through centuries and which holds biomechanical intrigue for both practitioners and researchers. The Taekwondo roundhouse kick and sine-wave punch, the Muay Thai clinch, the Capoeira martelo de negative kick or the Brazilian Jiu-Jitsu arm-bar. Each technique fulfils a purpose and was developed well before technology was available to provide kinematic insight. Although the sine-wave punch was found to be the most powerful punch in the Taekwondo techniques, the practitioner would only use it in the correct situations. What is more remarkable, is the continued discovery of human biomechanics and motion in these arts. With further advancements in technology and deeper analysis, we may uncover even further unknown details about the martial arts.

## Figures and Tables

**Table 1:** Fight Science martial arts punching comparison experiment.

Martial Arts Style	Punching Force (N)
Boxing	4417
Taekwondo	4079
Karate	3630
Kung Fu	2722

**Table 2:** Velocity results for Sine-wave punch and Reverse-step punch trials.

Punch Trial	Fist Velocity (m/s)	Bag Velocity (m/s)	Impact Force (N)
Sine-wave Punch 1	9.10	0.70	8834
Sine-wave Punch 2	9.84	0.71	6476
Sine-wave Punch 3	8.67	0.87	5342
Sine-wave Punch Mean	9.20	0.76	6884
Reverse-step Punch 1	9.56	0.51	4775
Reverse-step Punch 2	8.21	0.68	5041
Reverse-step Punch 3	7.78	0.59	5348
Reverse-step Punch Mean	8.51	0.59	5055
Overall Punch Trials Mean	8.86	0.68	5970

**Table 3:** Momentum and impulse results for Sine-wave punch and Reverse-step Punch Trials.

Punch Trial	Bag Velocity (m/s)	Bag Momentum (kg·m/s)	Impulse (N)	Effective Body Mass (kg)
Sine-wave Punch 1	0.70	36.40	238.5	4.00
Sine-wave Punch 2	0.71	36.92	254.6	3.75
Sine-wave Punch 3	0.87	45.24	312.0	5.21
Sine-wave Punch Mean	0.76	39.52	272.6	4.29
Reverse-step Punch 1	0.51	26.52	182.9	2.77
Reverse-step Punch 2	0.68	35.36	243.9	4.30
Reverse-step Punch 3	0.59	30.68	211.6	3.94
Reverse-step Punch Mean	0.59	30.85	212.6	3.60
Overall Punch Trials Mean	0.68	35.19	242.6	3.95

**Table 4:** Martial Arts styles comparison impact force results.

Punch Type	Impact Force (N)
Hapkido Punch 1	5219
Hapkido Punch 2	6669
Hapkido Punch Mean	5944
Wushu Punch 1	5827
Wushu Punch 2	6669
Wushu Punch Mean	6248

**Table 5:** Martial arts punching techniques mean force to weight ratio

Practitioner	Technique	Mean Impact Force (N)	Weight (kg)	Force to Weight
Taekwondo	Sine-wave punch	6884	125	55:1
Taekwondo	Reverse-step punch	5055	125	41:1
Hapkido	Right cross	5944	85	70:1
Wushu	Right cross	6248	76	82:1

**Table 6:** Impulse and Impact Force results for Sine-wave punch and Reverse-step punch trials.

Punch Trial	Impact Force (N)	Impulse Force (N)
Sine-wave Punch 1	8834	239
Sine-wave Punch 2	6476	255
Sine-wave Punch 3	5342	312
Sine-wave Punch Mean	6884	273
Reverse-step Punch 1	4775	274
Reverse-step Punch 2	5041	244
Reverse-step Punch 3	5348	212
Reverse-step Punch Mean	5055	213
Overall Punch Trials Mean	5970	243

## Data Availability

The data presented in this study are available on request from the corresponding author.

## Abbreviations

The following abbreviations are used in this manuscript:

WTF	World Taekwondo Federation
ITF	International Taekwondo Federation
IMU	Inertial Measurement Unit
EMG	Electromyography
FPS	Frames per second
